# Comparison of measures of insulin sensitivity in early-lactation dairy goats

**DOI:** 10.3168/jdsc.2021-0095

**Published:** 2021-06-19

**Authors:** F. Zamuner, A.W.N. Cameron, B.J. Leury, K. DiGiacomo

**Affiliations:** 1 Faculty of Veterinary and Agricultural Sciences, The University of Melbourne, Parkville, Victoria 3010, Australia; 2 Meredith Dairy Pty Ltd., Meredith, Victoria 3333, Australia

## Abstract

This experiment aimed to investigate the correlations between surrogate indices of insulin resistance (IR)—namely, the homeostasis model of IR, the quantitative insulin sensitivity check index, and the revised quantitative insulin sensitivity check index—and measures of IR obtained from an intravenous glucose tolerance test (IVGTT) performed in early-lactation dairy goats. Saanen goats (n = 26) with varying levels of milk production (1.7–4.8 kg/d) were selected and underwent an IVGTT on 43 ± 0.7 d postpartum (mean ± standard deviation). Data from the IVGTT were fitted in the minimal model to calculate parameters of glucose–insulin dynamics such as insulin sensitivity index and acute insulin response to glucose. Surrogate indices were computed using the average of the IVGTT basal samples. Correlation analysis revealed no relationship between surrogate indices of IR and measures of IR derived from the IVGTT (e.g., insulin sensitivity index, glucose clearance rate, glucose area under the curve). Therefore, our results suggest that surrogate indices of IR are not suitable for assessing the insulin sensitivity of peripheral tissue in early-lactation goats.

## Highlights

• Surrogate indices of IR and measures of insulin sensitivity derived from the IVGTT were not correlated.
• Surrogate indices were moderately correlated with measures of insulin secretion.
• Surrogate indices of IR are not indicated for assessing peripheral tissue insulin sensitivity in early-lactation goats.

## Graphical Abstract

**Figure:**
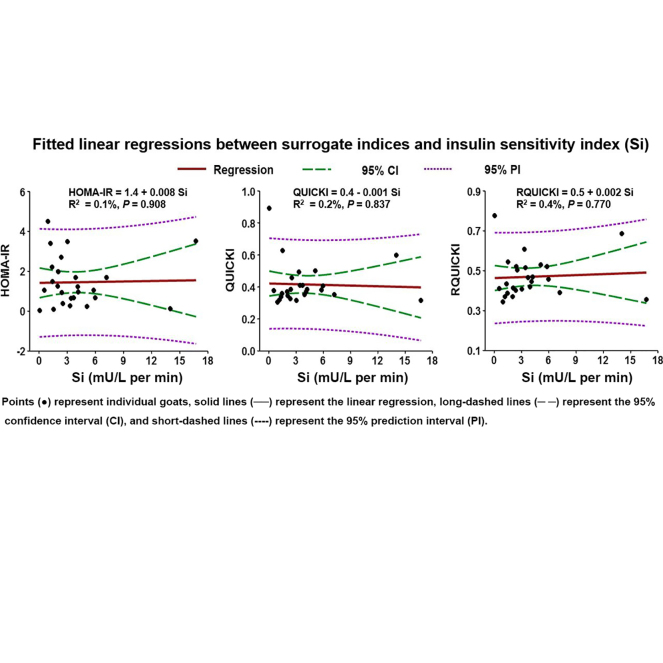
**Summary:** This experiment aimed to examine the association between surrogate indices of insulin resistance (IR)—namely, the homeostasis model of IR (HOMA-IR), quantitative insulin sensitivity check index (QUICKI), and revised quantitative insulin sensitivity check index (RQUICKI)—and measures of IR obtained from an intravenous glucose tolerance test (IVGTT) performed in early-lactation dairy goats. Despite a moderately strong correlation between surrogate indices of IR and insulin area under the curve, we found no significant relationship between surrogate indices and measures of reduced insulin sensitivity, such as glucose clearance rate, glucose area under the curve, and insulin sensitivity index. Thus, our results suggest that surrogate indices of IR are not suitable for assessing the insulin sensitivity of peripheral tissue in early-lactation goats.

Insulin resistance (**IR**) is defined as a state in which normal concentrations of insulin produce a less-than-normal biological response in insulin-sensitive tissues (e.g., liver, muscle, adipose tissues) to the metabolic actions of insulin (Kahn, 1978; De Koster and Opsomer, 2013). Although peripartum IR is an evolutionary adaptation of mammals to prioritize nutrient supply to placental and mammary cells over maternal reserves, thereby favoring offspring survival (Kampmann et al., 2019), increased IR is the key cause of metabolic disorders in periparturient humans (Miao et al., 2020). Also, several authors have found associations between IR and health disorders in transition cows and ewes (Opsomer et al., 1999; Duehlmeier et al., 2013; Xu et al., 2014). Thus, the development of methods to quantify IR has been widely explored.

The hyperinsulinemic-euglycemic clamp (**HEC**) test described by DeFronzo et al. (1979) is regarded as the gold-standard method to determine IR in humans and animals (De Koster and Opsomer, 2013). The HEC test allows for the evaluation of peripheral insulin sensitivity and responsiveness because it measures the capacity of insulin to promote glucose utilization under hyperinsulinemic conditions (Singh and Saxena, 2010; De Koster and Opsomer, 2013). However, this method is time consuming, laborious, expensive, and difficult to apply in large-scale investigations (Singh and Saxena, 2010; Cincović et al., 2018). The intravenous glucose tolerance test (**IVGTT**) is a cost-effective alternative to the HEC test. Using insulin and glucose measurements obtained during an IVGTT, it is possible to estimate insulin sensitivity and secretion with reasonable accuracy (Hahn et al., 2011). However, the fact that glucose disappearance during the IVGTT is the result of glucose uptake by both insulin-sensitive and insulin-insensitive tissues (e.g., mammary gland, placenta, kidneys) might preclude the appropriate interpretation of results in lactating animals (De Koster and Opsomer, 2013; De Koster et al., 2016). Data from the IVGTT can be fitted to a minimal model (**MINMOD**) to estimate several parameters of glucose–insulin kinetics with greater accuracy (Boston et al., 2003).

Nevertheless, the need for cheaper and less laborious methods to estimate IR (compared with HEC test and IVGTT) has driven the development of several surrogate indices for IR in humans, most of which rely on the assumption that fasting glucose and insulin levels represent a basal steady-state condition (Singh and Saxena, 2010). The surrogate indices most frequently used are the homeostasis model of IR (**HOMA-IR**), the quantitative insulin sensitivity check index (**QUICKI**), and the revised quantitative insulin sensitivity check index (**RQUICKI**; Singh and Saxena, 2010; De Koster and Opsomer, 2013).

Although such surrogate indices have been used to estimate IR in dairy cows (Holtenius and Holtenius, 2007; Mann et al., 2016; Marinković et al., 2019) and dairy goats (Cai et al., 2018), recent attempts to validate the use of surrogate indices for the determination of IR in dairy cows have reported conflicting results (De Koster et al., 2016; Mann et al., 2016; Cincović et al., 2017). Although the applicability of surrogate indices for the determination of IR is also of interest in the caprine species, this has not been demonstrated in goats. Therefore, this study aimed to examine the association between surrogate indices (HOMA-IR, QUICKI, and RQUICKI) and measures obtained from IVGTT, and parameters derived from minimal model analyses of IVGTT, in early-lactation dairy goats.

All experimental procedures were approved by the Faculty of Veterinary and Agricultural Sciences Animal Ethics and Welfare Committee of The University of Melbourne, Australia (no. 1714287.1). This experiment was conducted at Meredith Dairy commercial farm (Meredith, VIC, Australia; 37°50′S, 144°04′E). The experiment used 26 clinically healthy Saanen dairy goats, in second or third parity, that kidded in March 2018 (early autumn). Further details on animals, diet, and measurements (e.g., BCS, BW, ECM) and a detailed description of the IVGTT protocol are presented in Zamuner et al. (2020a). Briefly, goats were kept in individual stalls (15 m^2^) from 11 to 45 ± 4.2 d postpartum (mean ± SD) and ad libitum fed a TMR once daily at around 0900 h.

On the morning of d 42 (wk 6) after an overnight fast, a 14-gauge, 3.25-inch angiocath catheter (BD) was inserted into the jugular vein. A 22-cm plastic catheter extension with a Luer lock (Heidelberg extension tubing; B. Braun) prefilled with heparinized saline (50 U/L) was secured to the catheter. During blood sampling procedures, the catheter was flushed with heparinized saline (25 U/L) immediately after every blood sample collection. On d 43 (after overnight fasting) a 50% glucose solution was administered intravenously at 0.3 g of glucose/kg of BW, and blood samples were collected at −30, −15, −1, 2, 3, 4, 5, 6, 8, 10, 12, 14, 16, 18, 20, 25, 30, 35, 40, 60, 90, 120, 150, 180, 210, 220, and 240 min relative to glucose infusion. All blood samples were collected into vacuum tubes (10 mL) coated with lithium heparin (BD), immediately placed on ice, and centrifuged (1,250 × *g*, 4°C) for 12 min within 1 h after collection. Isolated plasma was stored at −20°C until analysis.

Plasma fatty acids concentrations were measured using a commercially available kit (NEFA-C ACS-ACOD method, modified per the methods of Johnson and Peters, 1993; Wako Pure Chemical Industries Ltd.). Plasma glucose concentrations were measured using a commercially available kit (Infinity Glucose Oxidase Liquid, Thermo Fisher Scientific). Plasma insulin concentrations were measured using an RIA kit (Porcine Insulin cat. no. PI-12K, Millipore Corp.) validated in goats by Maia-Nogueira (2015). Assay sensitivity (limit of detection) ranged between 0.4 and 0.5 mU/L for insulin. Every sample was assayed in duplicate. Intra- and interassay coefficients of variation, respectively, were <7.0% and <3.5% for glucose, <6.0% and <3.0% for fatty acids, and <10% and <4.1% for insulin.

Basal concentrations for insulin, glucose, and fatty acids were calculated as the mean concentration of the 3 blood samples taken before the glucose infusion. Plasma insulin, glucose, and fatty acid responses were analyzed for the area under the curve (**AUC**) using a linear trapezoidal summation between successive pairs of metabolite concentrations after correcting for baseline concentrations. Clearance rates (**CR**), time to reach half-life (**T_1/2_**), and basal concentrations (**T_b_**) were calculated per Pires et al. (2007) and surrogate indices were calculated per Singh and Saxena (2010) using the following equations:

CR_glucose_ = [ln (m*M* at 2 min) − ln (m*M* at 60 min)/(60 − 2 min)] × 100

CR_insulin_ = [ln (mIU/L at 20 min) − ln (mIU/L at 60 min)/(60 − 20 min)] × 100

T_1/2_ = [ln (2)/CR] × 100

T_b_ = {[ln (2 min) − ln (240 min)]/CR} × 100

HOMA-IR = [basal glucose (m*M*) × basal insulin (mIU/L)]/22.5

QUICKI = 1/[log glucose (mg/dL) + log insulin (mIU/L)]

RQUICKI = 1/[log glucose (mg/dL) + log insulin (mIU/L) + log fatty acids (m*M*)]

Performance traits were calculated using the following equations:

ECM = milk (kg) × 0.3246 + (0.1356 × % of fat + 0.0704 × % of protein) (Cai et al., 2018)

DMI (% of BW) = DMI (kg/d)/BW (kg)

Indices of glucose–insulin dynamics were calculated using MINMOD Millennium software (MINMOD Inc.; Boston et al., 2003), and the derived variables of interest were insulin sensitivity index (**Si**), which refers to the capacity of insulin to promote glucose uptake; glucose effectiveness (**Sg**), which refers to insulin-independent glucose disappearance rate; and acute insulin response to glucose (**AIR**), which addresses the adequacy of insulin secretion in response to a glucose bolus. Statistical analyses were performed using Minitab software (version 18.1; Minitab Inc.). Spearman rho correlation was used to examine relationships between variables of interest. Statistical significance was declared at *P* < 0.05, and values of *P* < 0.1 were considered a trend toward significance. Insulin concentration is given in milliunits per liter, and concentrations of glucose and fatty acids are given in millimolar unless stated otherwise.

Descriptive statistics for indices derived from the IVGTT and from the minimal model , surrogate indices, and performance traits are presented in Table 1. Basal concentrations and AUC for glucose, insulin, and fatty acids were within the range reported in healthy dairy goats (Cai et al., 2018). The large coefficient of variation observed for basal insulin and fatty acids and for insulin and fatty acids AUC could reflect factors such as the varying level of milk production (1.7–4.8 kg/d) and the potential varying energy status associated with it, but also could be due to the inherent constraints of reaching a basal steady state in ruminants.

**Table 1:** Descriptive statistics of measures of insulin sensitivity derived from the intravenous glucose tolerance test, surrogate indices for insulin resistance, and performance traits in early-lactation dairy goats (n = 26)

Variable^1^	Mean	SEM	SD	CV
Glucose				
Basal (m*M*)	3.2	0.10	0.49	15
CR_2–30_ (%/min)	2.3	0.10	0.49	21
T_1/2_ (min)	31	1.2	6.1	20
T_b_ (min)	78	3.5	17.6	23
AUC (m*M*/min)	359	23.3	118.6	33
Insulin				
Basal (mU/L)	9.3	1.43	7.31	79
CR_20–60_ (%/min)	2.3	0.27	1.4	59
T_1/2_ (min)	62	5.0	25.3	41
AUC (mU/L per min)	5,066	611.0	3,117.0	62
Fatty acids				
Basal (m*M*)	0.5	0.07	0.37	69
AUC (m*M*/min)	−9.9	5.63	28.72	−290
HOMA-IR	1.4	0.24	1.23	86
QUICKI	0.42	0.025	0.127	30
RQUICKI	0.47	0.020	0.103	22
Si (mU/L per min)	3.9	0.78	3.90	99
Sg ×10^3^ (m*M*/min)	16.5	0.97	4.84	29
AIR (mU/L per min)	455	47.3	236.5	52
ECM (kg/d)	3.2	0.18	0.89	28
DMI (%)	3.1	0.14	0.71	23
BW (kg)	67	1.6	7.9	12
BCS	2.3	0.10	0.53	22

1 CR = clearance rate; T_1/2_ and T_b_ = time to reach half-life and basal concentration after the glucose infusion, respectively; AUC = area under the response curve during the first 120 min of the intravenous glucose tolerance test; HOMA-IR = homeostasis model of insulin resistance; QUICKI = quantitative insulin sensitivity check index; RQUICKI = revised quantitative insulin sensitivity check index; Si = insulin sensitivity; Sg = glucose effectiveness; AIR = acute insulin response to glucose.

In human medicine, insulin-resistant individuals are expected to have an elevated insulin concentration, which can be accompanied by increased glucose concentration, increased fatty acids, or both (Wilcox, 2005). Thus, high HOMA-IR or low QUICKI and RQUICKI values are indicative of a higher degree of IR in humans (Singh and Saxena, 2010). Also, reduced glucose CR and increased glucose AUC, T_1/2_, and T_b_ are thought to be indicative of impaired insulin sensitivity in humans and ruminants (Muniyappa et al., 2008; De Koster and Opsomer, 2013). However, correlations presented in Table 2 show no significant relationship between surrogate indices of IR and glucose CR, AUC, T_1/2_, and T_b_ or between surrogate indices and Si. These findings indicate that the use of such surrogate indices is not suitable to assess the sensitivity of the peripheral tissues to insulin in early-lactation dairy goats.

**Table 2:** Spearman rho correlations between measures of insulin sensitivity derived from the intravenous glucose tolerance test, surrogate indices of insulin resistance,^1^ and performance traits in early-lactation dairy goats (n = 26)

Item^2^	HOMA-IR	QUICKI	RQUICKI
Glucose			
Basal (m*M*)	0.76***	−0.76***	−0.60**
CR_2–30_ (%/min)	0.06^NS^	−0.06^NS^	0.22^NS^
T_1/2_ (min)	−0.33^NS^	0.33^NS^	0.11^NS^
T_b_ (min)	−0.15^NS^	0.15^NS^	−0.19^NS^
AUC (m*M*/min)	−0.13^NS^	0.13^NS^	−0.14^NS^
Insulin			
Basal (mU/L)	0.99***	−0.99***	−0.89***
CR_20–60_ (%/min)	−0.26^NS^	0.26^NS^	0.33†
T_1/2_ (min)	−0.21^NS^	0.21^NS^	0.32^NS^
AUC (mU/L per min)	0.53**	−0.53**	−0.56**
Fatty acids			
Basal (m*M*)	−0.46*	0.46*	0.09^NS^
AUC (m*M*/min)	0.36†	−0.36†	−0.08^NS^
Si (mU/L per min)	−0.18^NS^	0.18^NS^	0.25^NS^
Sg ×10^3^ (m*M*/min)	0.05^NS^	−0.05^NS^	0.12^NS^
AIR (mU/L per min)	0.55**	−0.55**	−0.51**
ECM (kg)	−0.52**	0.52**	0.56**
DMI (% of BW)	−0.54**	0.54**	0.60**
BCS	0.51**	−0.51**	−0.48*

1 HOMA-IR = homeostasis model of insulin resistance; QUICKI = quantitative insulin sensitivity check index; RQUICKI = revised quantitative insulin sensitivity check index.

2 CR = clearance rate; T_1/2_ and T_b_ = time to reach half-life and basal concentration after the glucose infusion, respectively; AUC = area under the response curve during the first 120 min of the intravenous glucose tolerance test; Si = insulin sensitivity; Sg = glucose effectiveness; AIR = acute insulin response to glucose.

* *P* < 0.05

** *P* < 0.01

*** *P* < 0.001; †*P* < 0.1; ^NS^*P* > 0.1.

The observed lack of correlation between surrogate indices of IR and measures of insulin sensitivity derived from the IVGTT and minimal model may be explained by the inherent differences in metabolism between humans and ruminants. For instance, in ruminants, it is impossible to reach a basal steady-state condition without going through prolonged starvation, which in turn would cause changes to insulin, glucose, and fatty acids concentrations unrelated to the state of IR of the animal (De Koster et al., 2017). Additionally, some common features of the periparturient period in ruminants are negative energy balance, decreased insulin and glucose concentration, and increased fatty acids concentration (De Koster and Opsomer, 2013). Such physiological differences make it difficult to extrapolate to ruminants the interpretation of measures of IR in human medicine (De Koster et al., 2016; Cincović et al., 2017).

For instance, we observed a positive correlation between RQUICKI and ECM (r = 0.56), which is similar to previous observations in dairy goats (r = 0.45, *P* < 0.001; Zamuner et al., 2020b). By applying the medical interpretation of RQUICKI values, one may be misled to believe that increasing milk production is associated with decreasing degree of IR, even though the most plausible explanation is that the observed positive relationship was due to decreased insulin and increased fatty acids concentration in animals of higher milk production, as demonstrated in Zamuner et al. (2020a). Indeed, several studies in dairy cows have suggested that RQUICKI is a better reflection of energy status than of IR itself (Schoenberg and Overton, 2011; Cincović et al., 2017; Hasegawa et al., 2019). Furthermore, comparisons between studies in goats and cows suggest that RQUICKI values are usually lower in postpartum cows compared with antepartum cows (Cincović et al., 2014; Marinković et al., 2019), whereas a retrospective analysis of data from our previous studies showed that RQUICKI values were lower in antepartum goats than in postpartum goats (0.43 vs. 0.49, *P* < 0.001, data not shown; Zamuner et al., 2020b) and lower in low-yielding goats than in high-yielding goats in early lactation (0.42 vs. 0.50; *P* = 0.01, data not shown; Zamuner et al., 2020a).

The differences between studies could be related to interspecies differences in the magnitude of changes in the fatty acids:insulin ratio between postpartum and antepartum animals, which might have a large effect on absolute RQUICKI values. For instance, Marinković et al. (2019) and Cincović et al. (2014) reported a significant decrease in insulin concentrations (~35%) but an approximate 250% increase in fatty acids concentrations in postpartum cows. Conversely, in Zamuner et al. (2020b), we observed that the increase in fatty acids concentration in postpartum goats (+188%) was accompanied by a more pronounced decrease in insulin concentration (−312%) compared with antepartum goats. Similarly, in Zamuner et al. (2020a), we observed that greater fatty acids concentration in high-yielding goats (+20%) was accompanied by a much lower insulin concentration (−130%) compared with low-yielding goats.Schoenberg and Overton (2011) pointed out the weaknesses of using RQUICKI to measure IR in ruminants, suggesting that interpretation of results between cows of different metabolic status or stage of lactation should be done with caution. Apparently, the same principle can be applied to comparisons between goats and cows. Therefore, due to the lack of information on dairy goats, it is rather difficult to compare our results with those reported in the literature. Further research is needed to determine the potential use of surrogate indices of IR to measure IR in dairy goats.

In humans, the pancreas compensates for reduced insulin action in peripheral tissues by upregulating insulin secretion (De Koster and Opsomer, 2013). In the present study, we observed a negative correlation between Si and insulin AUC (r = −0.59, *P* = 0.002) and between Si and insulin AIR (r = −0.45, *P* = 0.023). At first glance, the direction and strength of these correlations could be interpreted as compensatory hypersecretion of insulin in response to lower insulin sensitivity. However, these results should be interpreted with caution because in Zamuner et al. (2020a) we found no difference in peripheral tissue response to insulin between goats of different basal insulin levels (5.6 vs. 12.9 mU/L, *P* = 0.008). Therefore, the observed large interindividual variations in insulin production and secretion could be attributed to genetic differences, as has been demonstrated in humans (Hansen et al., 2020), or to differences in milk production and energy status (Zamuner et al., 2020a). Nevertheless, the reasons for the present findings remain speculative.

Several authors have reported a negative association between fatty acids concentration and pancreatic insulin secretion in dairy cows (De Koster and Opsomer, 2013; Cincović et al., 2018; Hasegawa et al., 2019). For the influence of fatty acids on glucose–insulin kinetics (or vice versa) in dairy goats, our results are somewhat ambiguous. In the present study, the increased basal concentration of fatty acids was associated with reduced basal insulin, reduced glucose CR, and increased glucose AUC, T_1/2_, and T_b_ (r = −0.47, −0.61, 0.65, 0.48, and 0.61, respectively; *P* < 0.01), suggesting that increased lipid mobilization was associated with increasing IR. Nevertheless, we found no significant correlation between basal fatty acids and insulin AUC or between basal fatty acids and any minimal model–derived measures of IR (Si, Sg, AIR). Therefore, more detailed investigations are needed to determine the role, if any, of lipid mobilization in glucose–insulin kinetics in dairy goats.

Considering the lack of significant correlations between the surrogate indices of IR and measures of insulin sensitivity derived from the IVGTT and the minimal model, we suggest that the studied surrogate indices of IR are not suitable for assessing insulin sensitivity of peripheral tissue in early-lactation goats. Nevertheless, given the relatively small set of animals used in the present study and the limited literature on insulin production, secretion, and sensitivity in dairy goats, further research is needed to confirm, or to expand on, the potential use of surrogate indices of IR to measure differences in IR, or in energy status, in early-lactation goats. Moreover, RQUICKI values were strongly correlated with basal insulin and moderately correlated with insulin AUC and AIR, but no significant correlation was found between RQUICKI and basal fatty acids or fatty acids AUC.
